# The Climate and Topography of Aiken, South Carolina, in Their Relation to Phthisis

**Published:** 1864-11

**Authors:** E. S. Gaillard

**Affiliations:** Surgeon and Medical Inspector.


ART. II.—*The Climate and Topography of Aiken, South Carolina, in their Relation to Phthisis.* By E. S. GAILLARD, Surgeon and Medical Inspector.

As our ports are all comparatively blockaded, and egress is inhumanly denied to the invalid, it is proper that the profession should meet such an emergency as satisfactorily as it has done many others which have arisen during the progress of this war.

Phthisis, that disease over which science mourns, because of her inability to afford relief, claims its victims as remorselessly now, as it has ever done, in times past. The hecatombs that have been annually sacrificed upon the altar of this insatiable Moloch are sadly increased by the incidents and exposures of the field.

In the treatment of this disease, all well-informed physicians of the present day appreciate the wisdom of the Thane of Cawdor, in the well known injunction to his professional adviser, and seek in the obscure, yet magic influences of climate, that balmy elixir which Pharmacy has, so far, failed to reveal.

Until comparatively recent years, the influences and adaptation of climate and topography, in their relations to Phthisis, have never received the adequate investigation of competent observers. Patients have been sent indifferently and indiscriminately to the dry, cold atmosphere of Spitzbergen, or to the warm, moist air of Bermuda and Jamaica; to the temperate climate of Madeira, Florida and the Mediterranean, or to the dry and warm atmosphere of Cairo and Sierra Leone.

Independently of the trying circumstances with which war has surrounded us, it is well that this subject should receive thorough attention, but now, that the consumptive is barbarously denied access to his usual resorts, it is especially necessary that some proper and appropriate provision be made for his physical welfare.

It is on this account that the climate and topography of Aiken, South Carolina, are brought to the attention and consideration of the profession

Aiken is situated in the Northwestern part of Barnwell District, South Carolina, and quite near the dividing line, between Barnwell and Edgefield Districts—more accurately described, its latitude is 33° 32′ North, and its longitude 81° 34′ West.

It is situated on the South Carolina Railroad, one hundred and twenty miles from Charleston, S. C., and sixteen miles from Augusta, Georgia.

The village is built near the centre of an elevated plateau, or table land, possessing an area of about twenty square miles. The elevation of this plateau is 565 feet above the level of the sea, and about 300 feet above the Savannah river, at its nearest point.

The country immediately adjacent is drained by Shaw's creek and Horse creek, with several smaller streams, emptying some into the Savannah, and some into the Edisto river; this drainage is thorough and complete.

The character of the soil is sandy, with a subsoil of red clay; the stratum of superficial soil being from six to ten feet deep, unless at the immediate site of the village, where the substratum of clay crops out. The sand is of the white variety, silex entering largely into its composition. This soil is exceedingly dry, water not being found at a less distance than from eighty to one hundred and twenty-five feet below the surface.

The water is of a superior character, being transparently clear, with a temperature varying from 62° to 65° Fahrenheit; it is generally impregnated with the Salts of Iron and Magnesia, but not sufficiently so to affect its taste or to render it deleterious to the invalid.

The annual rain fall at this point, as tested by the rain-guage, is usually, about thirty-seven inches, or a monthly average of three inches; the heaviest uniform fall being in the months of June, July and August, and the smallest fall in Autumn.

The earliest frost usually occurs from the 10th to the 15th of November, and the latest from the 1st to the 10th of April; the average duration of the period without frost being from two hundred to two hundred and twenty-five days; or about two-thirds of the year. This fact is deserving of special attention.

The mean annual temperature is from 50° to 54° Fahrenheit; the mean temperature of the Winter months being from 42° to 46° F.; that of the Spring months 58° F.; that of the Summer months 77° F., and that of Autumn 62° F. Attention is directed to the very gradual and equable variation in these temperatures, there being a marked absence of sudden changes, and the variations in temperature being strictly in accordance with the respective seasons.

The extremes in temperature for one year (which is a fair type of the seasons of other years) are as follows: January, 60°—40° Fahrenheit; February, 76°—33° F.; March, 82°—24° F.; April, 73°—28° F.; May, 86°—50° F.; June, 92°—66° F.; July, 86°—64° F.; August, 92°—69° F.; September, 90°—53° F.; October, 78°—40° F.; November, 83°—19° F.; December, 74°—31° F.

The prevailing winds are from the South and Southwest.

The dew point is invariably low. This hygrometrical condition of the atmosphere is here characteristic. The ordinary long moss (Tilandsia) of the Gulf States, as has been frequently tried by experiment, will not grow here. The Cryptogamous plants are feebly represented, and those only grow that are usually found flourishing in dry atmospheres. Very few lichens are to be seen; on the tops of the highest and dryest hills a few specimens are to be found. The grape flourishes in great profusion and abundance, and it is very unusual for the atmosphere to be sufficiently damp to cause any injury to this delicate fruit.

The forest growth is of the dwarf oak and the yellow pine; the atmosphere is decidedly terebinthinate.

Endemics are unknown here, and epidemics rare. The country is entirely free from malarial diseases.

This village, for a series of years has been the resort of consumptives from many sections of the United States, and their testimony, in regard to the climate, is very satisfactory.

The difference in this climate and that of the seaboard is so marked that many persons suffering from pulmonary trouble have, after residing at Aiken, been unable, with impunity, to return to their homes upon the coast.

The climate and water, together, have produced very conspicuous results in the health of those suffering from gastric and intestinal complications.

Having thus given these physical and meteorological facts, it is proper that a few reasons should be furnished for considering this village as peculiarly adapted for the home or retreat of the consumptive.

Bernard, Ingenhouze, Cruikshank and others have satisfactorily demonstrated the important fact that the skin is, physiologically, the chief supplement in respiration. Becquerel, Rodier, Todd, and other observers, have shown that this supplementary action of the skin is peculiarly and vitally important in Phthisis; corroborating views, long since expressed by Laennec, and a few years since interestingly brought to the notice of the profession by Dr. Lee, of London.

Sir James Clark, Ancell and others similarly interested, have demonstrated the interesting truth that the supplementary action of the skin is in a direct ratio with the hygrometric condition of the atmosphere. Edwards has confirmed these experiments, and states that in a moist atmosphere, the supplementary action of the skin is at its minimum. (Lee.)

Phillips, Tulloch and Forry testify that it is not only true that the action of the skin is least in moist atmospheres, but that this fact is usually manifested, irrespective of atmospheric temperature.

Interesting tables have been prepared by Fourcault, and also by Ancell, showing that where dryness of atmosphere exists, irrespective of temperature, there is a marked increase in cutaneous transpiration, and that where this condition is found, Phthisis is seldom observed.

It is a familiar fact with the profession that in the dry and hot atmospheres of the Cape of Good Hope, Egypt and Sierra Leonne, Phthisis is exceedingly rare; that it is equally rare in the temperate air of Australia, the Mediterranean coast, Madeira, Florida and the slopes of the Pacific, whilst in the dry, cold regions of Iceland, Greenland, Sweden, etc., this disease is comparatively unknown.

The happy effect of dry atmospheres are conspicuously manifested in the South of Spain, France and Italy, (in sections of these countries unvisited by the Mistral, found so frequently prevailing,) and throughout the entire extent of this isothermal line of Humboldt.

Dr. Hay, who accompanied Kane's Arctic Expedition, states that among the Esquimeaux tuberculosis and all forms of scrofula were unknown.

It will be observed, that at all of these places, dryness is the prevailing and distinguishing characteristic of the atmosphere.

In direct contrast with these facts, it is well known to all who have investigated this subject, that moist atmospheres, irrespective of temperature, have usually a baneful effect in most cases of Phthisis; this is especially true in those cases representing the second and third stages of the disease. The statistics of Nova Scotia, Holland, England and Jamaica have long since demonstrated this truth.

In analyzing the oral and written testimony with reference to Phthisis, in its relation to climate and topography, the whole subject resolves itself into a few well marked and easily recognized divisions.

There is the dry and cold climate which is usually both prophylactic and curative in its influences. Spitzbergen, Sweden, and Minnesota, etc., may be accepted as representatives of this class.

The dry and temperate climate found on the Pacific slopes, in Middle Florida, at Aiken, South Carolina, Boulogne, Nice, St. Remo, Dieppe and Southern Spain. This air is also prophylactic and curative, and is especially adapted to the largest class of cases observed.

The dry and warm climate, as found at Hyeres, Menton,

Naples, etc.; the only practical difference between this air and that to which allusion has last been made, is that a dry and warm atmosphere is frequently found to be too exciting.

The dry and hot climate peculiar to Cairo, Algiers and Sierra Leonne. This air is desirable where stimulation is required and is well borne.

The moist and cold climate; that of Wales, Scotland, Nova Scotia and New England; provocative of the disease and prejudicial in its influences.

The moist and temperate climate, as found on the Isle of Wight, at Pau, Pisa and Eastern Florida, and in Holland. This air is beneficial, though not in so marked a degree as that which is dry and temperate; it is not as often applicable.

The moist and warm climate: Bermuda, Jamaica, Madeira and Sicily.

The moist and hot climate, as found in Cuba and most of the West India Islands. The air at most of the localities specified in the two last divisions is usually beneficial in incipient cases of Phthisis, but is almost invariably baneful, if not fatal, to those in the last stages of the disease.

It will be observed, in this summary, that no air is more frequently appropriate and beneficial than that of Aiken, South Carolina, resembling, as it does, that of Nice and St. Remo, which are regarded at the present time with more favor, perhaps, than any other sections of Europe.

The elevation of the country at and around Aiken is also a material advantage to consumptives, as it is a well known fact that respiration, in its depth and volume, is in a direct ratio with atmospheric agitation. Edwards and others have made several interesting experiments for the establishment of this truth. (Lee.)

Aiken possesses another great virtue in the important fact that the consumptive residing there can, with impunity, exercise in the open air, throughout the year. M. M. Lombard, L'Espine, Dujat and others have made interesting tables, showing the disastrous results invariably ensuing, when consumptives reside in those sections, requiring prolonged residence within doors. The researches of these observers corroborate the position, first advanced by M. Carriere, that hybernation and mortality in Phthisis are in a direct ratio. The soil at this place is particularly well adapted for safe exercise throughout the winter.

The important physiological fact being accepted, that the skin is, in its action, supplementary to the lungs, that there is, so to speak, a cutaneous, as well as a pulmonic respiration, it is evident, when, from disease, there is a deficiency or inefficiency in the action of the lungs, that it is vitally necessary to develope and stimulate the functions of the skin. Nothing so happily or successfully accomplishes this object as residence and active exercise in a dry, equable and temperate climate. It should be remembered that the beneficial effect of climate, in arresting cases of consumption, is particularly successful, in proportion as the disease is in its incipient stage.

There are eligible spots in and around this village which can be advantageously purchased, and the facilities for building are all that could be desired. The soil is especially adapted to gardening and horticulture, and with very little care, an abundant supply of vegetables, and very superior fruit may be obtained. The grape and peach orchards in this vicinity are especially rich and valuable.

The distinguishing characteristics of the Aiken climate then, are its peculiar dryness of the atmosphere, its temperate and equable temperature, its freedom from sudden and violent atmospheric changes, an absence of frost for two-thirds of the year, its freedom from endemics and malarial diseases, and the general prevalence of soft Southern and Southwesterly breezes.

These important and interesting facts in regard to this locality, in connection with its dry and porous soil, rendering exercise at all times practicable; its excellent water and abundant fruit; cheerful and refined society; its remarkable elevation; its facility of access and removal from all crowded centres of population, with their irregular hours and inseparable excitements, render Aiken especially adapted for the home of the consumptive.

